# Donkey whey protein and peptides regulate gut microbiota community and physiological functions of D‐galactose‐induced aging mice

**DOI:** 10.1002/fsn3.3111

**Published:** 2022-10-21

**Authors:** Xueyan Zhou, Xiaojing Tian, Li Song, Li Luo, Zhongren Ma, Fumei Zhang

**Affiliations:** ^1^ College of Life Science and Engineering Northwest Minzu University Lanzhou China; ^2^ China‐Malaysia National Joint Laboratory, Biomedical Research Center Northwest Minzu University Lanzhou China; ^3^ Gannan Research Institute of Yak Milk Hezuo China; ^4^ The Department of Medicine Northwest Minzu University Lanzhou China

**Keywords:** aging, donkey whey hydrolysate, donkey whey protein, gut microbiota

## Abstract

The prolongation of life span has attracted more and more attention in the current world. Gut microbiota is considered one of the most critical elements and is essential in regulating life span and quality. The effects of donkey whey protein (DWP) and donkey whey hydrolysate (DWPP) on physiological functions and gut microbiota of D‐galactose‐induced aging mice were investigated to find new strategies for resisting aging. Our results showed that DWP and DWPP could increase the body weight gain velocity, superoxide dismutase (SOD) activity, and thymus index, whereas decrease the level of reactive oxygen species (ROS) and malondialdehyde (MDA), and improve the aging of the body in the liver congestion, oozy draw focal sclerosis of chronic inflammation. The effects of medium and high concentrations of DWP and low and medium concentrations of DWPP were the same as the vitamin C (Vc)‐positive control group. It was found that both DWP and DWPP could change α‐diversity; the relative abundance of *Lactobacillus* increased, whereas the relative abundance of *Helicobacter* and *Stenotrophomonas* decreased after being treated with DWP and DWPP. The correlation between intestinal microflora and physiological indexes showed that chao1, ACE, and observed species indexes in the α index were positively correlated with weight gain velocity, SOD activity, and thymus index. The relative abundance of *Lactobacillus* was positively correlated with SOD and thymus index but negatively correlated with MDA. The relative abundance of *Stenotrophomonas* was opposite to that of *Lactobacillus*. The *Anaerobiospirillum*, *Fusobacterium*, and *Dubosiella* had a significant positive correlation with the weight gain velocity. The study provided a deeper more profound understanding of the potential use of DWP and DWPP in senescence delays.

## Graphical abstract

DWP and DWPP could increase the body weight growth rate, antioxidant activity, immune function, and against chronic hepatitis damage in aging mice.

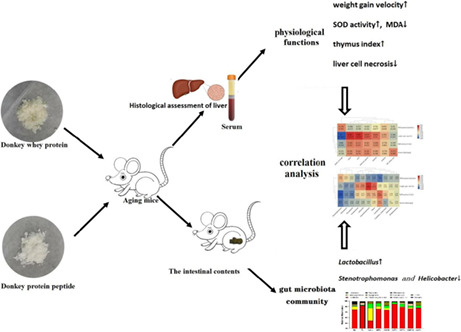

## 1 INTRODUCTION

The prolongation of life span has attracted more and more attention due to the increase in economy and life quality. However, it is still a global challenge to improve the health span of people (Dzau et al., 2020; Partridge et al., 2018). Aging, which is the time‐dependent functional decline affecting most living organisms, has attracted curiosity and excited imagination throughout the history of humankind (López‐Otín et al., 2013). Aging is a complicated process that is influenced by a variety of factors such as the gastrointestinal/digestive system, immune system, nervous system, metabolic system, various biological signaling pathways, programmed cell death (PCD), and environmental factors (Adriansjach et al., 2020). A better understanding of aging is necessary for developing novel approaches to prolong the life span and increase life quality, especially for the elders.

Among the factors affecting aging, the gut microbiota are considered one of the most critical elements and plays an essential role in regulating life span and quality (O'Toole & Jeffery, 2015). The microbiota composition of the gut changes with age, and alterations in this composition affect human health (Odamaki et al., 2016; Xu et al., 2019). A large body of microorganisms resided in the gut, approximately between 10^13^ and 10^14^ (Liu et al., 2020). The composition of gut microbiota has been shaped in early childhood, becomes mature by the age of 3 years, and stays stable over the life span (Fouhy et al., 2019). The 16S ribosomal DNA‐sequencing data of fecal samples indicated that the structure and diversity of gut microbiota got remarkable changes in older adults compared with young settings (Kim & Jazwinski, 2018). It was found that the proportion of *bacteroides*, *clostridia*, and total *lactobacilli* declined, while the population of *fusobacteria*, *streptococci*, and *staphylococci* increased in older adults compared with the younger group (Woodmansey et al., 2004). The changes in structure and diversity of gut microbiota often influence innate immunity's homeostasis, further affecting body functions and health (O'Toole & Jeffery, 2015). Herein, the investigations toward the relationship between gut microbiota and aging will open up new opportunities for discovering new strategies to slow down aging.

Donkey milk has been recognized for nutritional and therapeutic purposes since ancient times (Cunsolo et al., 2017). Donkey milk is an excellent replacer of cow milk for infants who suffer from cow milk protein allergy (CMPA), and its composition (e.g., the contents of both lactose and protein) is quite similar to human milk (Martini et al., 2018; Souroullas et al., 2018). Emerging evidence shows that donkey milk has certain physiological functions, including high tolerability, antimicrobial, and anticancer activities (Li et al., 2020). However, the effects of donkey milk on gut microbiota remain unclear and need further investigation.

Therefore, the present work aimed to investigate the effects of DWP and DWPP on the composition and abundance of gut microbiota in D‐galactose‐induced aging mice. The microbial community and relative abundance were evaluated by 16S rRNA gene sequencing, and the relationship between gut microbiota and physiological and functional features was predicted by correlation analysis. The results are expected to improve our understanding of the association between gut microbiota changes and aging, which may provide practical information for discovering the underlying mechanism of aging and novel approaches to improving aging problems.

## 2 MATERIALS AND METHODS

### 2.1 Materials

Donkey milk was obtained from the local farm (Lanzhou, China), taken to the laboratory within 1 h, and stored at −20°C for further use. All other chemicals and reagents used in this work were provided locally and were of analytical grade.

### 2.2 Methods

#### 2.2.1 Preparation of DWP and DWPP

For the DWP preparation, donkey milk was centrifuged with a centrifuge (Heraeus Multigauge X1R, Thermo Fisher) at 4700 *g* (4°C) for 30 min. The upper fraction (milk fat) and a down fraction (milk whey and granule) could be visualized after centrifugation. Upper milk fat was discarded, and the pH of left skim milk was adjusted to 4.6 with 1 M HCl, followed by incubating in the water bath (40°C) for 20 min. Then, skim milk was centrifuged at 12,000 *g* at 4°C for 20 min, and supper milk whey (MW) was collected and then lyophilized. The MW powder was stored at 4°C until further use.

For DWPP preparation, 0.5 g DWP was dissolved in pure water and the final concentration was adjusted to 30 mg/ml. Then, it was incubated at 90°C for 5 min, followed by cooling down to 50°C by stirring with a magnetic stirrer, and adjusted pH to 7.0. The concentration of neutral protease was carefully adjusted to 4000 U/g. Hydrolysis lasted for 5 h; during the hydrolysis, pH was kept to 7.0. After hydrolysis, the reaction system was heated to 100°C for 10 min to inactivate the protease. Then, the reaction system was centrifuged at 15,000 *g* for 10 min, and the upper fraction (DWPP) was collected, then lyophilized and stored in the fridge (4°C) for the following experiments.

#### 2.2.2 Chemical composition of DWP and DWPP

The Kjeldahl method determined the protein content (AOAC, 1995). The conversion factor used to convert the Kjeldahl nitrogen value (N) to protein amount was 6.25. Results were expressed as g per 100 g dry weight (g/100 g DW). Fat and lactose content were determined by the method described in GB5009.6‐2016 (National Standard of the People's Republic of China: national standard for food safety, determination of fat in food, China, 2016) and GB25595‐2018 (National Standard of the People's Republic of China: national standard for food safety, determination of lactose in food, China, 2018), respectively. The pH‐stat method was used to examine the hydrolysis efficiency of DWP.

#### 2.2.3 Animal experiments

All (total of 65) healthy female Kunming mice (age 6 weeks, 25–30 g body weight) were provided by the Experimental Animal Center of the Institute of Genetics and Developmental Biology of the Chinese Academy of Sciences (Lanzhou, China). The mice were kept under standard laboratory conditions and housed in an environmentally controlled room (24 ± 1°C) with 55 ± 10% relative humidity in a 12 h light/dark automatic lighting cycle. They could access the standard pellet diet and drink water ad libitum throughout the study. The Research Ethics Review Committee approved all animal and biological experiments of the College of Life Science and Engineering, Northwest Minzu University (Lanzhou, China); ethical approval number: xbmu‐sm‐2019002. Mice were subcutaneously injected with 10% D‐galactose solution (1.25 g/kg/day) for 6 weeks to establish aging models. Subsequently, 54 of 65 animals were randomly divided into nine groups: non‐treatment control group (NS), negative control (Aging), positive control (Vitamin C, Vc), low‐, middle‐, and high‐concentration donkey milk whey protein (DWP) treatment group (DWPL, DWPM, and DWPH, respectively), and low‐, middle‐, high‐concentration donkey milk whey peptide (DWPP) treatment (DWPPL, DWPPM, and DWPPH, respectively). All groups except for NS were sequentially injected with 10% D‐galactose solution (1.25 g/kg/day) by subcutaneous injection. In parallel, different doses of vitamin C, DWP and DWPP were intragastrically supplemented to the aging models for 7 weeks. All experimental designs are presented in Table 1. Physiological criteria, including body and thymus weight, were determined following the methods described by Farhangi et al. (2017) and Kong et al. (2018). The weight gain velocity (Sié et al., 2021; Stahr et al., 2021) was calculated with the formula below: when: *N* > 2, *M* = *N*‐2; *N* = 1, *M* = 0,

$$\text{weight}\;\text{gain}\;\text{velocity}=\frac{\text{weight}\;\mathrm{at}\;N\;\text{weeks}-\text{weight}\;\mathrm{at}\;M\;\text{weeks}}{\text{weight}\;\mathrm{at}\;M\;\text{weeks}}\times 100\%.$$

**TABLE 1:** Details of different treatments in different groups.

Groups	Treatment	
	Subcutaneous injection	Intragastric administration
NS	0.9% NaCl 112.5 mg/kg/day	0.9% NaCl 0.5 ml/mouse/day
Aging	10% D‐galactose 1.25 g/kg/day	0.9% NaCl 0.5 ml/mouse/day
Vc	10% D‐galactose 1.25 g/kg/day	Vitamin C 300 mg/kg/day
DWPL	10% D‐galactose 1.25 g/kg/day	DWP 100 mg/kg/day
DWPM	10% D‐galactose 1.25 g/kg/day	DWP 200 mg/kg/day
DWPH	10% D‐galactose 1.25 g/kg/day	DWP 400 mg/kg/day
DWPPL	10% D‐galactose 1.25 g/kg/day	DWPP 100 mg/kg/day/day
DWPPM	10% D‐galactose 1.25 g/kg/day	DWPP 200 mg/kg/day
DWPPH	10% D‐galactose 1.25 g/kg/day	DWPP 400 mg/kg/day

*Note*: NS: Non‐treatment control; Aging: negative control; Vc: vitamin C positive control; DWPL, DWPM, and DWPH represent low‐, middle‐, and high‐concentration DWP intervention group, respectively. DWPPL, DWPPM, and DWPPH represent low‐, middle‐, and high‐concentration DWPP intervention group, respectively.

Next‐generation sequencing (NGS) of the 16S rRNA gene amplicons method was utilized to check the gut microbiota in the intestinal contents collected from three mice (they were selected from every group randomly).

#### 2.2.4 Assays of SOD and MDA

The superoxide dismutase (SOD) activity and malondialdehyde (MDA) content were determined with the commercial kit manufactured by Nanjing Jiangcheng Bioengineering Institute (Nanjing, China) according to the instructions.

The sample and reagents were added to the tubes and incubated at 37°C for 40 min. And then, a chromogenic reagent was added and mixed. After being placed at room temperature for 10 min, the absorbance was recorded by a Microplate Reader (Bio‐Rad 550). As for MDA content, the sample and reagents were added into the tubes and incubated at 95°C for 40 min. After cooling to room temperature, they were centrifuged at 1360 *g* for 10 min, and the absorbance of the sample was measured with a Microplate Reader (Dai et al., 2008).

#### 2.2.5 ROS lever determination

ROS level was determined according to the method described in the previous study (Zhong et al., 2019). One hundred milligram of liver tissue was mixed with 1 ml buffer and homogenated using a grinder. Then, the mixture was centrifuged at 4°C for 10 min and 190 μl of supernatant was incubated with 10 μl of BBoxiProbe O13 ROS probe (BestBio) in a 96‐well plate at 37°C in the dark for 25 min. ROS levels were quantified by a fluorescence microplate reader (Bio‐Rad 550) with an excitation wavelength of 488 nm and an emission wavelength of 606 nm.

#### 2.2.6 Histological assessment of liver

Hematoxylin and eosin (H&E) staining of the liver was performed according to the method described in the previous study (Kong et al., 2018). Liver tissue was collected and fixed in 4% paraformaldehyde (v/v) for 24 h. Then, samples were dehydrated in ascending grades of alcohol, then embedded in paraffin, and finally, sectioned at a thickness of 5 μm. H&E stained these samples for routine examination of these tissues, and pictures were recorded by light microscope (Olympus CX31).

#### 2.2.7 Analysis of the intestinal bacteria of mice via 16S rRNA gene sequencing

About 2 g of stool samples in the ileum of the mice were aseptically collected, and then a MoBio Powersoil DNA extraction kit (MoBio) was utilized to extract the total DNA in fecal samples. The quality of DNA was detected by running an agarose gel (1%). DNA was quantified using NanoDrop™ 2000/2000c Spectrophotometers (Thermo Scientific™). Qualified DNA samples (A260/280 value of approximately 2.0, DNA amount ≥500 ng) was fragmented through ultrasonication (approximately 300 bp in length), and a V3–V4 region library of the 16S rRNA gene was constructed based on the DNA templates by using TransStart Fastpfu DNA Polymerase (TransGen Biotech). The primer sequences for the polymerase chain reaction were as follows: the 515 (5′‐GTGCCAGCMGCCGCGGTAA‐3′) and 806 (5′‐GGACTACHVGGGTWTCTAAT‐3′). The polymerase chain reaction assay was performed using the following program: initial denaturation at 95°C for 10 min, followed by 30 cycles of 95°C for 15 s, annealing at 60°C for 15 s, and extension at 72°C for 30 s; final extension at 72°C for 5 min. Three technical duplicates with negative controls for each gene were conducted under the same conditions. The target fragments of DNA were collected by GeneJET RNA Purification kit (Thermo Scientific) according to the manufacturer's instructions. The Qubit2.0 (Life Tech) and Agilent 2100 Bioanalyzer (Agilent) were used to determine the densities of the collected fragments and execute quality control, respectively. The efficiency of the adapters was evaluated by qPCR. The sequencing process was accomplished using the IonS5TMXL system (Thermo Fisher Scientific).

#### 2.2.8 Sequence data analysis

The raw data were filtered using Cutadapt (V1.9.1) quality‐controlled process to remove barcode, sequences of 5′‐end primer mismatch base number >1, sequences with length ≤150 bp, sequences with a continuous equal number of bases >8, and ambiguous sequences. Then, the reads were submitted to the Silva database (https://www.arb‐silva.de/) to identify and delete chimeric and nontarget region sequences using UCHIME (version 4.1). The sequences were classified using the Uparse software (version 7.0.1001; http://drive5.com/uparse/) and classified into different operational taxonomic units (OTUs) based on the sequence similarity cut‐off value (i.e., 97%). The abundance (reads number) of OTUs in each sample was calculated and the OTUs containing more than two reads were used for further analysis. The alpha‐diversity analysis (e.g., observed OTUs, abundance‐based coverage, Chao, Shannon, Simpson, coverage, and rarefaction curve) and beta‐diversity analysis (e.g., nonmetric multidimensional scaling, principal component analysis, and principal coordinates analysis) were analyzed by QIIME (v1.8.0) software. The unweighted UniFrac distance of beta‐diversity index, SILVA rRNA database (http://www.arb‐silva.de/) on Mothur website (http://www.mothur.org/wiki/RDP_reference_files), and Ribosomal Database Project (RDP) classifier (80% confidence) were used to calculate the principal coordinates analysis (PCoA), the annotation of the OTUs, and OTUs relative abundances (from phylum to species level), respectively.

#### 2.2.9 Statistical analysis

The results were presented as mean ± standard deviation. A one‐way analysis of variance (ANOVA) test was used for analysis. Duncan's multiple‐range test was performed in multiple comparisons using IBM SPSS Statistics 22.0 (SPSS Inc.). The significant difference was determined at *p* < .05.

## 3 RESULTS

### 3.1 Chemical composition of DWP and DWPP

The chemical composition and hydrolysis efficiency were determined and results are shown. The protein content was 10.4 ± 0.6 g/100 g, lactose content was 78.9 ± 0.8 g/100 g, and fat content was 0.396 ± 0.14 g/100 g. The hydrolysis efficiency was 10.2%.

### 3.2 Effects of DWP and DWPP on the physiology of D‐galactose‐induced aging in mice

**FIGURE 1:**
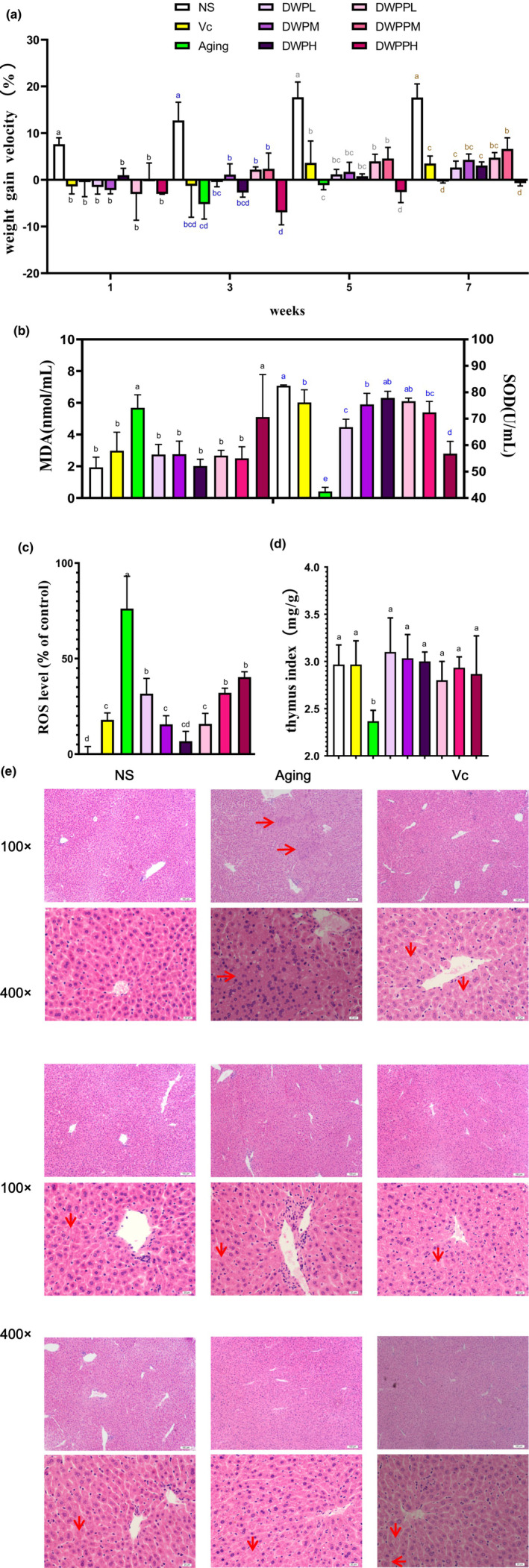
Effects of DWP and DWPP on the physiology of D‐galactose‐induced aging in mice. (a) DWP and DWPP intervention in aging mice body weight gain velocity changes; (b) DWP and DWPP intervention in the aging changes of SOD activity and MDA content; (c) DWP and DWPP intervention in the aging changes of the ROS lever in the liver; (d) DWP and DWPP intervention in the aging changes of thymus index; and (e) DWP and DWPP intervention in aging mice liver tissue morphology. In the picture, “→” is the liver tissue with high coagulation necrosis and cirrhosis. “←” is the serous exudate gathered in the hepatic sinusoid space. “↓” is necrotic or apoptotic liver cells. It can be seen that the nuclei of the liver cells are fragmented, the nuclear membrane disappears, and the nucleolus is dissolved, and some nuclei shrink. NS: nontreatment control; Aging: negative control; Vc: positive control; DWPL, DWPM, and DWPH represent low‐, middle‐, and high‐concentration DWP intervention group, respectively; DWPPL, DWPPM, and DWPPH represent low‐, middle‐, and high‐concentration DWPP intervention group, respectively. Histograms on top of which the same letter appears to represent means that are not statistically different; different letters identify means that are significantly different (*p* < .05).

It is attractive to investigate the effects of DWP and DWPP on the physiology of D‐galactose‐induced aging mice, since donkey milk has been demonstrated to play an essential role in maintaining body functions. As shown in Figure 1, at the end of the 3rd, 5th, and 7th weeks, the weight gain velocity of the intervention groups except DWPPH group was higher than that of the aging group, and the changes in DWPH, DWPPL, and DWPPM groups were similar to or even better than that of the Vc group (DWPPM groups with Vc groups, *p* < .05; Figure 1a). MDA and SOD are commonly used antioxidant activity indexes, and the thymus index is a commonly used immune function index. MDA values in the intervention groups except DWPPH were statistically different from those in the aging group, while no significant differences were found between the NS and Vc groups. SOD value also increased, and DWPH and DWPPL groups returned to normal, DWPM and DWPPM groups and Vc group had no statistical difference (Figure 1b). In addition, the ROS level was tested and results are shown in Figure 1c. The ROS level increased by 76.2 times in the aging group in comparison with the control group. The ROS level in the intervention groups was significantly lower than in the aging group, indicating that donkey whey powder and its hydrolysates showed promising antioxidant activity in D‐galactose aging mice. The thymus index in all the intervention groups was significantly higher than the aging group, but not from the NS and Vc groups, indicating that donkey whey powder and its hydrolysates can significantly improve the immune function of D‐galactose aging mice (Figure 1d). In contrast, the hydrolysates of donkey whey powder with high concentration showed slight improvement, and there was no statistical difference between the aging groups.

As shown in Figure 1e, the histological assessment results showed the liver in D‐galactose‐induced aging mice exhibited atrophy status with hepatic sinus gap expansion, during which a large number of aggregate distribution serous effusions. This phenomenon might be due to the liver tissue edema, causes compression of liver cells, leading to different degrees of liver cell necrosis, mostly cash to liver cell nucleus disintegration and cellular structure disappearing entirely. The liver of the NS group exhibited a complete structure, clear morphology, orderly arrangement of hepatic cords, moderate size of hepatic sinusoidal lacunae, no exudation, edema, accumulation of heterochromic substances, high maturity of hepatic sinusoidal endothelial cells, the complete structure of hepatocytes, clear cell boundaries, complete nucleoli, and transparent nuclear membrane. The liver of Vc and the samples treated group (DWPL, DWPM, DWPH, DWPPL, and DWPPM) showed no abnormalities in liver tissue structure, except mild blood stasis in the central vein, and there was little difference among all groups. Most groups had scattered focal hepatocyte necrosis, coagulation, the disappearance of nuclear nucleoli, and destruction of the nuclear membrane, and scattered accumulation of hepatic sinusoidal endothelial cells in some regions, which might be organized nodules produced after liver injury. In the DWPPH group, atrophy of the hepatic cord and dilatation of hepatic sinusoidal space were also observed, during which a large amount of serous exudation was clustered and distributed, which might be due to the liver edema further causing the compression of hepatocytes in the liver.

### 3.3 Variations in richness and diversity of the microbial community

**FIGURE 2:**
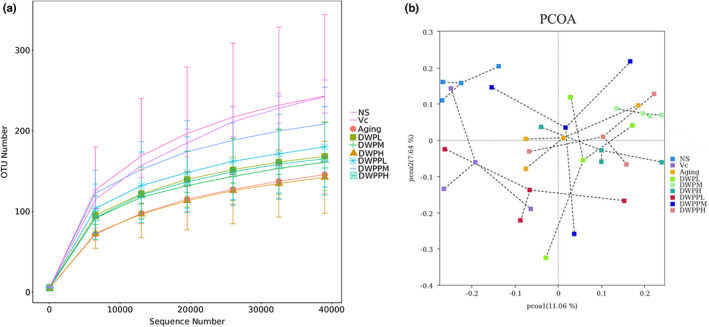
Variations in richness and diversity of the gut microbial community. (a) The rarefaction curve was constructed through random sampling of clean reads. (b) Principal coordinates analysis (PCoA) of beta diversity in the microbial communities in each group.

Firstly, the raw data were filtered and nonspecific amplified fragments and chimeras were removed; thus, quality control data were obtained (Table S1). It showed that the average clean reads obtained from each group ranged from 65,618 to 82,591. A rarefaction curve was constructed through random sampling of clean reads, which indicated that the curve tended to be flat (Figure 2a). It means that there are enough reads being included in the study. Principal component analysis (PCA) showed differences in the bacterial compositions of the nine groups (Figure 2b). These results showed that the DWPPL, normal, and Vc intervention groups were far away from the aging group, indicating that the intestinal flora richness and diversity were significantly different (*p* < .05).

**FIGURE 3:**
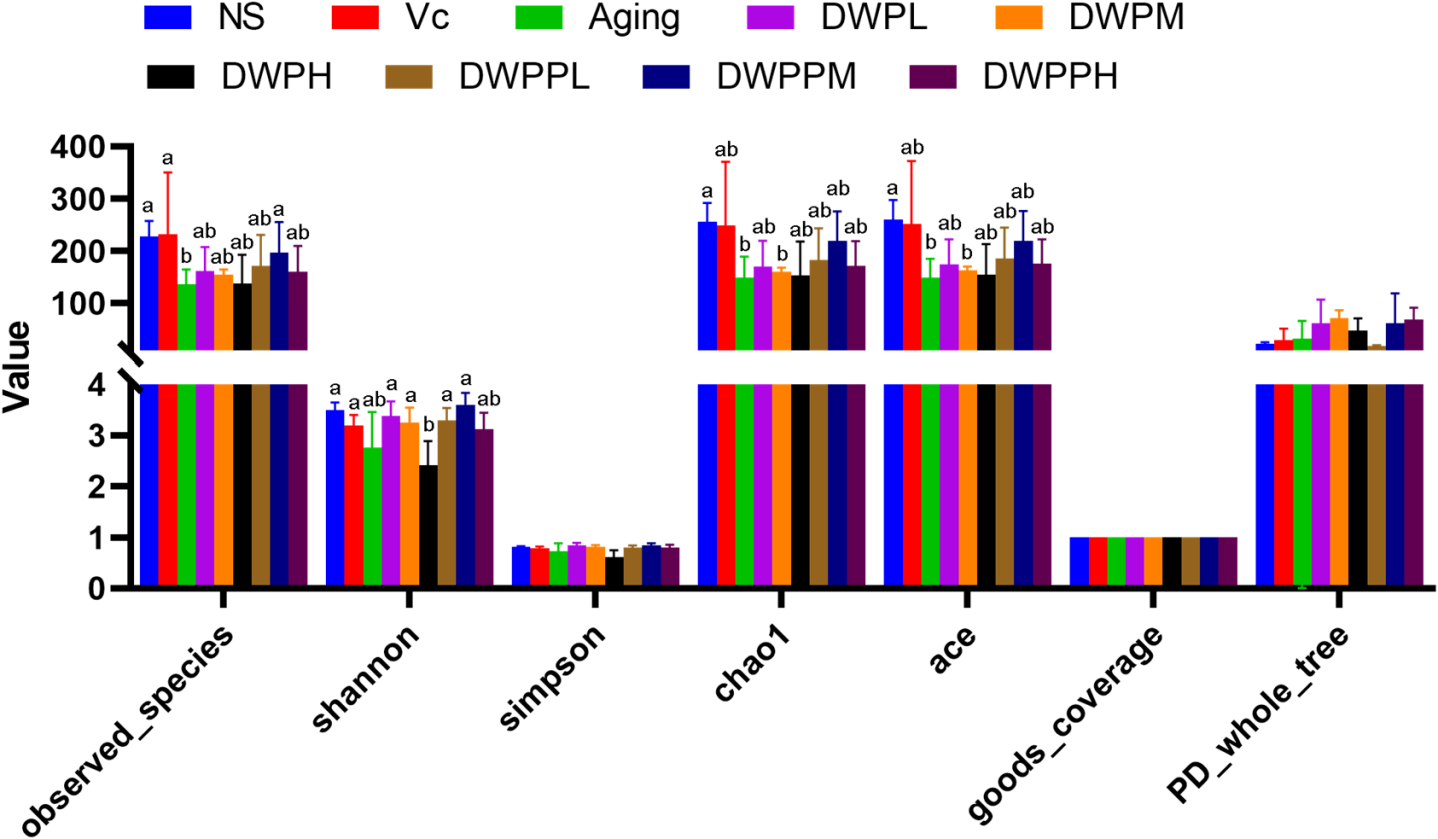
The alpha‐diversity analysis regarding microbial communities. The richness of the microbial community in aging is lowest compared with other groups according to alpha diversity indexes including Shannon index, Simpson index, chao1, and ace. Histograms on top of which the same letter appears represent means that are not statistically different; different letters identify means that are significantly different (*p* < .05). NS: nontreatment control; Aging: negative control; Vc: positive control; DWPL, DWPM, and DWPH represent low‐, middle‐, and high‐concentration DWP intervention group, respectively. DWPPL, DWPPM, and DWPPH represent low‐, middle‐, and high‐concentration DWPP intervention group, respectively.

Meanwhile, the results of the alpha‐diversity analysis showed that the richness of the microbial community in aging is lowest by comparison with other groups according to alpha‐diversity indexes, including Shannon index, Simpson index, chao1, and ace. For DWP‐ and DWPP‐treated groups, except DWPH and DWPPH, other groups had increased Shannon value, Simpson value, chao1 value, and ace value compared with aging (Figure 3); the results of DWPPM were closer to the Vc group. These results showed that besides the high‐concentration groups, the donkey whey and its hydrolysates significantly improved the intestinal flora in the senile state, especially DWPPM.

### 3.4 Effects of DWP and DWPP on gut microbiota community

**FIGURE 4:**
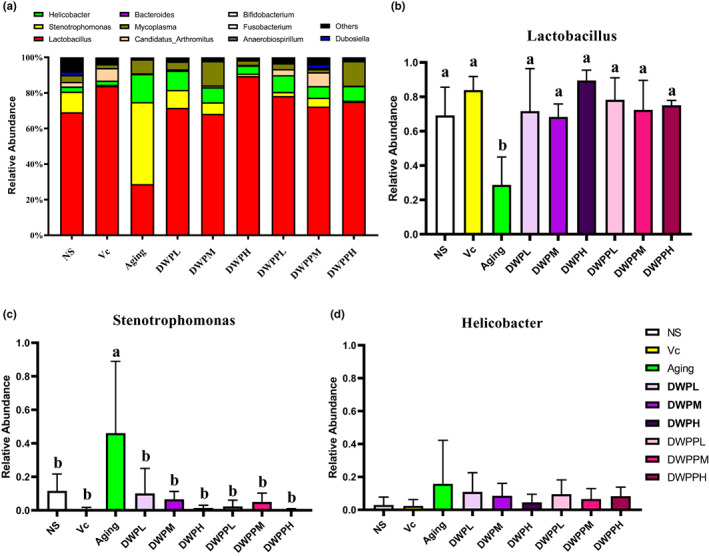
Effects of DWP and DWPP on gut microbiota community. (a) the relative abundance of the top 10 intestinal bacteria in all groups; (b) the relative abundance of *Lactobacillus* in different groups; (c) the relative abundance of *Stenotrophomonas* in different groups; and (d) the relative abundance of *Helicobacter* in different groups; NS: nontreatment control; Aging: negative control; Vc: positive control; DWPL, DWPM, and DWPH represent low‐, middle‐, and high‐concentration DWP treatment group, respectively. DWPPL, DWPPM, and DWPPH represent low‐, middle‐, and high‐concentration DWPP treatment group, respectively. Histograms on top of which the same letter appears represent means that are not statistically different; different letters identify means that are significantly different (*p* < .05).

Based on relative abundance data, *Lactobacillus*, *Helicobacter*, and *Stenotrophomonas* were the most abundant in all groups by comparison with other bacteria (Figure 4a). *Lactobacillus* in the NS group, Vc, and all the donkey whey and its hydrolysates intervention groups was significantly higher than that in the aging group (*p* < .05; Figure 4b), followed by *Stenotrophomonas*, whose relative abundance in the aging group was significantly higher than that in the other groups (*p* < .05; Figure 4c). There was no significant difference in the relative abundance of *Helicobacter* between groups. But aging was significantly higher than in the other groups, especially in the NS group, Vc group, and DWPH group (*p* = .143, .129, and .159, respectively) (Figure 4d). These results showed that DWP and DWPP have effects on the relative abundance of intestinal bacteria, and the detailed mechanism needs further investigation.

### 3.5 Correlation between alpha diversity and physiological characterizations

**FIGURE 5:**
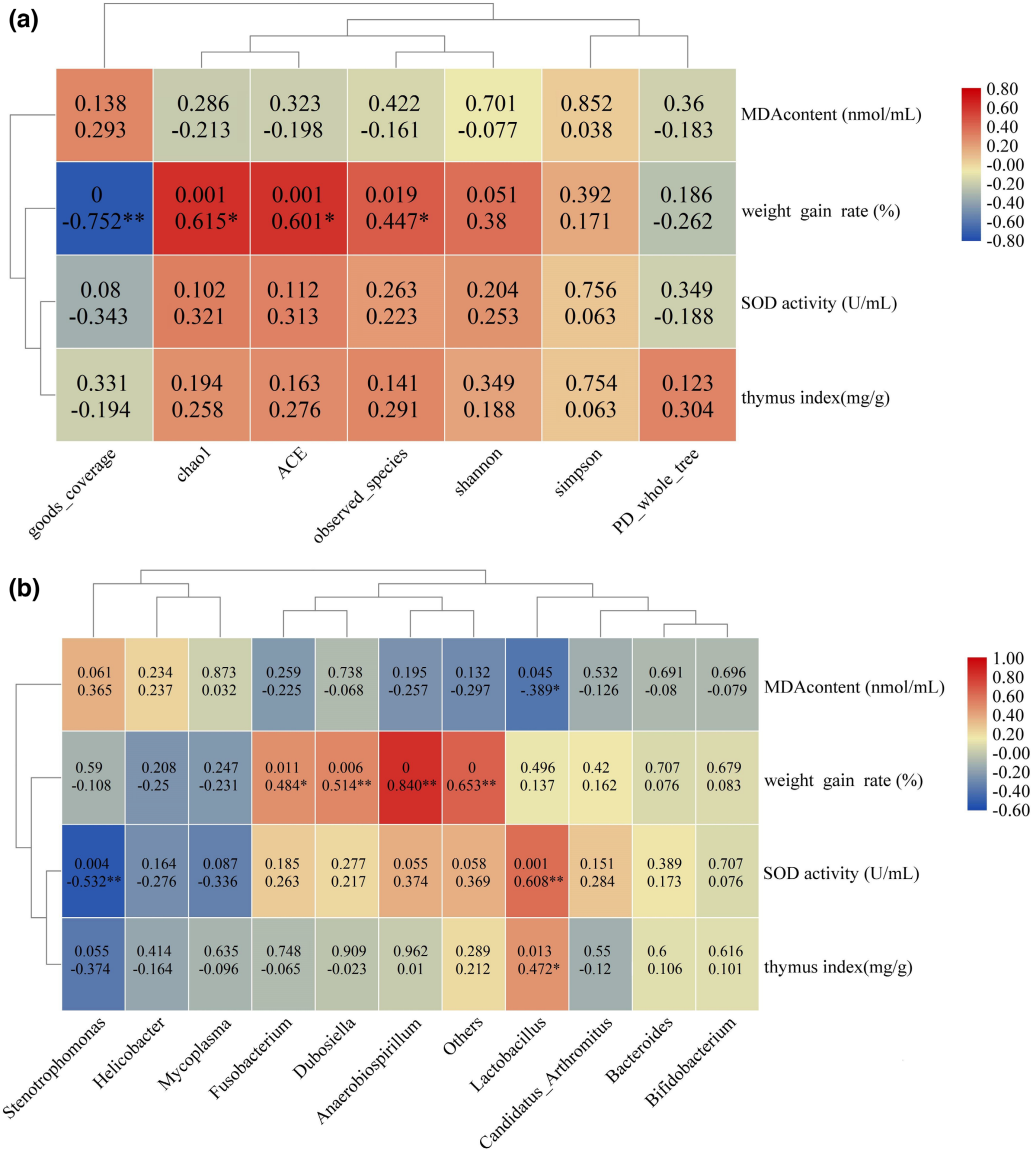
Correlation between alpha diversity and physiological characterizations. (a) Heatmap representing the correlation between α‐indexes and physiological characterizations; (b) heatmap representing the correlation between the abundance of dominant bacteria and physiological characterizations.

Exploring the relationship between alpha diversity of gut microbiota and physiological characterizations is worthwhile. Hence, a correlation analysis of the alpha diversity of intestinal bacteria and physiological parameters was established with Pearson analysis. As shown in Figure 5a, chao1, ace, and observed species were positively correlated with weight gain velocity, and their correlation indexes were .615 (*p* < .001), .601 (*p* < .001), and .449 (*p* < .05), respectively. Good coverage was negatively correlated with weight gain velocity. No significant correlation was found among other groups; it was found that SOD and thymus index and weight gain velocity have the same trend, while MDA was the opposite. The results showed that the physiological indexes were related to the diversity of intestinal flora. The relative abundance of *Lactobacillus* was positively correlated with SOD (correlation coefficient .608, *p* < .001) and thymus index (.472, *p* < .05), while negatively correlated with MDA (−.389, *p* < .05). The relative abundance of *Stenotrophomonas* and *Helicobacter* was opposite to that of *Lactobacillus*, and only *Stenotrophomonas* and SOD had statistical differences, while the others had no statistical significance. The body weight gain velocity was significantly positively correlated with that of *Anaerobiospirillum*, *Dubosiella*, and *Fusobacterium*. The results showed that antioxidant activity was positively correlated with the abundance of intestinal *Lactobacillus* and negatively correlated with *Stenotrophomonas*. However, it can be seen that SOD and thymus index and weight gain velocity have the same trend, while MDA has the opposite. The results showed that the physiological indexes were related to the diversity of intestinal flora (Figure 5b).

## 4 DISCUSSION

The present work investigated the effects of DWP and DWPP on gut microbiota and physiological functions in D‐galactose‐induced aging mice. It was found that both DWP and DWPP have significant effects on the body function and intestinal bacteria composition of aging Kunming mice. The alpha diversity of gut microbiota could be linked to the physiological functions, and dominant bacteria in the gut were moderately correlated with certain physiological functions.

The chemical composition analysis shows that the main components of whey powder are lactose and whey protein. Lactose is hydrolyzed into glucose and galactose, the former mainly provides energy for the body, while galactose binds to ceramide with a glycosidic bond to form galactose cerebral glycoside, which is involved in brain development (Sinha et al., 2007). It was found that the nutrition value of whey protein was much higher than other proteins (Sinha et al., 2007) as it contains rich essential amino acids and branched‐chain amino acids, as well as a balanced source of sulfur‐containing amino acids including isoleucine, leucine, and valine (Almeida et al., 2016). These essential amino acids play an important role in weight control, obesity prevention, various metabolic functions, and blood glucose homeostasis (Teixeira et al., 2019). In our study, the supplantation of DWP and DWPP with different concentrations can recover weight loss in the aging process, which was consistent with the nutritional supplement of lactose and whey protein.

Aging is accompanied by oxidative damage and changes in immune function. SOD is an important enzyme‐exerting antioxidative functions, and MDA is negatively correlated with antioxidative functions (Rasmussen & Suomi, 1989). The SOD activity improved, whereas the MDA content and ROS level decreased with the treatment of DWP and DWPP (Figure 1b,c), indicating that DWP and DWPP exhibited good antioxidant activity. Lactose has a terminal aldehyde group (HC=O) and can be oxidized to COO‐ (Considine & Frankish, 2013). Five millimeter lactose can not only reduce free radicals to 53.5% but it also plays a particular role in the formation of peroxide free radicals (Wehmeier & Mooradian, 1994). Whey protein is a complete protein rich in sulfur‐containing amino acids (Rastall et al., 2005) and branched amino acids, which can be used as a precursor of the antioxidant glutathione (GSH). Furthermore, whey products in cells (O'Keeffe & FitzGerald, 2014; Pyo et al., 2016), animals (Ashoush et al., 2013; J. Kim et al., 2013), and clinical (Sheikholeslami Vatani & Ahmadi Kani Golzar, 2012) were confirmed to have good antioxidant activity.

Nevertheless, many findings indicate that high protein intakes (more than 40% protein level) increase oxidative stress (Toyomizu et al., 1995) and decrease antioxidative activity in mice's digestive organs. This imbalance between the production of free radicals and the ability of the organism's natural protective mechanisms is due to the excessive oxidation of amino acids (Gu et al., 2008). Therefore, DWPPH could increase the thermogenic response, which is accompanied by a lower efficiency of food energy utilization, an increase in oxygen consumption, and impaired oxidative phosphorylation capacities. Thus, attention should be paid when using DWPP in treating aging patients.

The thymus index can reflect the body's immune function from the side. Subcutaneous injection of D‐galactose‐induced osmotic pressure and mitochondrial dysfunction in mice, and the formation of hydrogen peroxide reduced SOD level, resulting in oxidative stress and chronic inflammatory reaction (Azman & Zakaria, 2019). Therefore, the thymus index of mice in the aging group decreased significantly compared with other groups. Vc group and donkey whey powder and its hydrolysate intervention group can improve aging mice's thymus index and immune function. The results of liver staining showed that some liver cells in aging mice were focal necrosis due to inflammation. After the treatment of DWP and DWPP, the liver exhibited a complete structure, clear morphology, orderly arrangement of hepatic cords, complete structure of hepatocytes, and clear cell boundaries, which might be related to the fact that D‐galactose is mainly metabolized in the liver, leading to liver inflammation, and then liver structural and histological damage. Donkey whey powder and its hydrolysates at different concentrations could improve chronic inflammation in the liver of an aging organism; similar results were reported by Radic et al. (2019), and Fukawa et al. (2017) showed that the supplementation of whey protein could protect against chronic hepatitis damage.

The gut microbiota is crucial in regulating energy homeostasis and intake to control body functions (Wang et al., 2020; Yin et al., 2019). It was also demonstrated to be closely linked to aging (Adriansjach et al., 2020). Whey protein is essential in maintaining gut development and functions (Hahn et al., 2018). Firstly, lactose promotes gut health because the microbiota use it as a carbon source. Its fermentation is followed by carboxylic acids (lactic and butyric) that decrease the gut pH and create a nonfavorable medium for pathogenic bacteria (Kareb & Aïder, 2019). Secondly, whey protein also acts as a prebiotic in the intestinal tract, increasing the abundance of *Lactobacillus* in mice (Boscaini et al., 2020; Sánchez‐Moya et al., 2017). In humans, it was found that whey protein could enhance the diversity of gut microbiota to improve weight loss and/or alter appetite (Reimer et al., 2017). Different from cow milk, donkey milk was found to be closer to the composition of human milk, which makes it exert a particular function, such as improving life quality for infants allergic to cow milk (Sarti et al., 2019). Therefore, this experiment was conducted to study the effects of donkey whey powder and its hydrolysates on the intestinal microflora of aging mice, and to provide a theoretical basis for supplementing donkey whey powder in the aging population and slowing down aging.

High diversity of intestinal flora is associated with health, low diversity is associated with different diseases, and community diversity is negatively correlated with the vulnerability of the elderly (Jackson et al., 2016; Lloyd‐Price et al., 2016). The results of alpha diversity in this study (Figure 3) showed that Chao1 and ACE values of D‐galactose modeling aging mice decreased significantly compared with normal control mice, while the intestinal microflora diversity of positive control Vc group and donkey whey powder treated with different concentrations and its hydrolysates increased significantly compared with the aging group. The chao1, ace, and observed species were positive correlated with the weight gain velocity, SOD, and thymus index. Therefore, appropriately increasing the diversity of intestinal flora can promote the body to improve antioxidant capacity and resist aging. But more needs to be done to support this mechanism (Ma et al., 2021).

The structure of intestinal flora is affected by many factors, such as country, region, diet habits, gender, and disease, but it is still considered to be one of the crucial factors in determining longevity (Han et al., 2017; Santoro et al., 2018) and has been found that the gut microbiota structure of the elderly is very different from that of the young (Gruber & Kennedy, 2017; Park et al., 2015). In the elderly subjects, the abundance of Firmicutes decreased and the abundance of Bacteroidetes increased (Kumar et al., 2016), and the proportion of Firmicutes and Bacteroidetes significantly decreased (Mariat et al., 2009). The proportion of *Bifidobacterium* and *Lactobacillus*, which are healthy and beneficial, decreased significantly. In this study, different concentrations of donkey whey powder and its hydrolysates greatly influence the relative abundance of dominant bacteria in the intestinal flora of D‐galactose‐induced aging mice, such as *Lactobacillus*, *Stenotrophomonas*, and *Helicobacter*. Compared with other groups, MDA and SOD in the aging group prepared with D‐galactose were significantly increased, while the abundance of beneficial *Lactobacillus* in the intestinal tract was significantly decreased, while the harmful bacterial flora *Stenotrophomonas* and *Helicobacter* were significantly increased, indicating that the aging organism was in a state of oxidative stress and intestinal flora disorder. After the treatment with different concentrations of donkey whey powder and its hydrolysates, the serum MDA and SOD of the mice decreased significantly. At the same time, the abundance of beneficial *Lactobacillus* in the intestinal tract increased significantly, while the harmful bacteria of *Stenotrophomonas* and *Helicobacter* decreased significantly. It was found that the relative abundance of *Lactobacillus* was positively correlated with SOD and thymus index but negatively correlated with MDA. The relative abundance of *Stenotrophomonas* was opposite to that of *Lactobacillus*. At the same time, donkey whey powder and its hydrolysate improved chronic inflammation in the liver of aging organisms. These results are consistent with the theory of Kong et al. (2018). and Sagi et al. (2020). respectively, that the increase in the composition and abundance of *Lactobacillus* contributes to the improvement of oxidative stress in the body and chronic inflammation in the liver. These results suggest that donkey whey powder and its hydrolysates may regulate the composition and abundance of *Lactobacillus* in the intestinal flora of aging bodies, thus showing a better antioxidant effect and improving the chronic inflammatory response in the liver of aging bodies.

## 5 CONCLUSION

The present work explored the effects of donkey whey protein and peptides on the composition and abundance of gut microbiota in D‐galactose‐induced aging mice. The result showed that DWP and DWPP could change the alpha diversity and the relative abundance of gut microbiota in D‐galactose‐induced aging mice. DWP and DWPP can improve antioxidative activity. DWP and DWPP increase the relative abundance of *Lactobacillus* and decrease the relative abundance of *Stenotrophomonas* and *Helicobacter*. The concentration remarkably influences the effects of DWP and DWPP on gut microbiota and physiological functions in aging mice. The study highlights the importance of DWP and DWPP in improving the health status of aging settings and opens new opportunities for future advances in developing novel strategies that slow down aging.

## AUTHOR CONTRIBUTIONS

Data curation, Li Luo; Formal analysis, Xiaojing Tian; Investigation, Li Song; Methodology, Zhongren Ma; Project administration and Resources, Fumei Zhang; Software and Writing—original draft, Xueyan Zhou; All authors read and approved the final manuscript.

## CONFLICT OF INTEREST

The authors have no conflicts of interest to declare.

## Supporting information


Table S1

## Data Availability

Data will be made available on request.
