# Supplementary material for: Donkey whey protein and peptides regulate gut microbiota community and physiological functions of D‐galactose‐induced aging mice
Source: Food Sci Nutr. 2022 Oct 21;11(2):752–64. doi: 10.1002/fsn3.3111 (PMC9922154; doi:10.1002/fsn3.3111)
Supplement: Supplementary file 1 — Table S1 [file FSN3-11-752-s001.docx]

| #Sample_name | Raw_reads(#) | Clean_Reads(#) | Base(nt) | AvgLen(nt) | Q20 | GC% | Effective% |
| --- | --- | --- | --- | --- | --- | --- | --- |
| NS1 | 85258 | 80230 | 34131152 | 425 | 85.27 | 52.42 | 94.1 |
| NS2 | 82143 | 80105 | 33363550 | 416 | 82.4 | 50.7 | 97.52 |
| NS3 | 85694 | 80041 | 34230984 | 427 | 87.97 | 52.19 | 93.4 |
| Aging1 | 83258 | 80062 | 32926962 | 411 | 83.31 | 48.22 | 96.16 |
| Aging2 | 86822 | 80288 | 34089236 | 424 | 83.51 | 51.8 | 92.47 |
| Aging3 | 83455 | 80216 | 34312492 | 427 | 82.55 | 52.05 | 96.12 |
| Vc1 | 86899 | 80101 | 33718226 | 420 | 85.7 | 51.34 | 92.18 |
| Vc2 | 61732 | 56988 | 24087493 | 422 | 84.49 | 51.62 | 92.32 |
| Vc3 | 64547 | 59765 | 25152054 | 420 | 82.66 | 51.49 | 92.59 |
| DWPL1 | 84263 | 80062 | 33570287 | 419 | 82.64 | 51.15 | 95.01 |
| DWPL2 | 81888 | 80072 | 33881045 | 423 | 86.11 | 50.89 | 97.78 |
| DWPL3 | 84303 | 80276 | 32685741 | 407 | 85.11 | 49.06 | 95.22 |
| DWPM1 | 84568 | 80248 | 33870284 | 422 | 85.34 | 49.32 | 94.89 |
| DWPM2 | 82636 | 80151 | 34049761 | 424 | 85.08 | 50.35 | 96.99 |
| DWPM3 | 82655 | 80131 | 33836751 | 422 | 85.31 | 50.56 | 96.95 |
| DWPH1 | 83290 | 80162 | 33583856 | 418 | 86.33 | 50.48 | 96.24 |
| DWPH2 | 84821 | 80153 | 34205169 | 426 | 81.5 | 50.7 | 94.5 |
| DWPH3 | 82569 | 80160 | 34250795 | 427 | 80.57 | 50.95 | 97.08 |
| DWPPL1 | 83394 | 80177 | 32703327 | 407 | 86.34 | 49.13 | 96.14 |
| DWPPL2 | 86672 | 80018 | 33543413 | 419 | 85.7 | 49.85 | 92.32 |
| DWPPL3 | 89591 | 87579 | 36579758 | 417 | 85.69 | 50.64 | 97.75 |
| DWPPM1 | 82805 | 80144 | 33741727 | 421 | 84.57 | 50.86 | 96.79 |
| DWPPM2 | 82365 | 80178 | 33101358 | 412 | 84.1 | 50.74 | 97.34 |
| DWPPM3 | 88265 | 80230 | 34126138 | 425 | 85.16 | 51.3 | 90.9 |
| DWPPH1 | 85141 | 80021 | 33059211 | 413 | 86.98 | 49.87 | 93.99 |
| DWPPH2 | 83162 | 80055 | 33084578 | 413 | 83.52 | 46.76 | 96.26 |
| DWPPH3 | 86028 | 80139 | 33785781 | 421 | 86.01 | 51.05 | 93.15 |

supplementary table 1：Statistical table of each sample sequence
